# Supplementary material for: Predicting visual function by interpreting a neuronal wiring diagram
Source: Nature. 2024 Oct 2;634(8032):113–23. doi: 10.1038/s41586-024-07953-5 (PMC11446822; doi:10.1038/s41586-024-07953-5)
Supplement: Supplementary file 6 — CRF and ERF predictions for individual TmY4 and TmY9 cells. Analogous to Supplementary Data 3, but for TmY target types. Shown are the top four monosynaptic pathways, the strongest pathway passing through each of the top ten intermediary types (ranking from Extended Data Fig. 7), and the trisynaptic pathway Tm1–TmY–Dm3–TmY (see the section entitled Prediction of spatial normalization). [file 41586_2024_7953_MOESM6_ESM.zip › DataS4/TmY9q/720575940630737679.pdf]

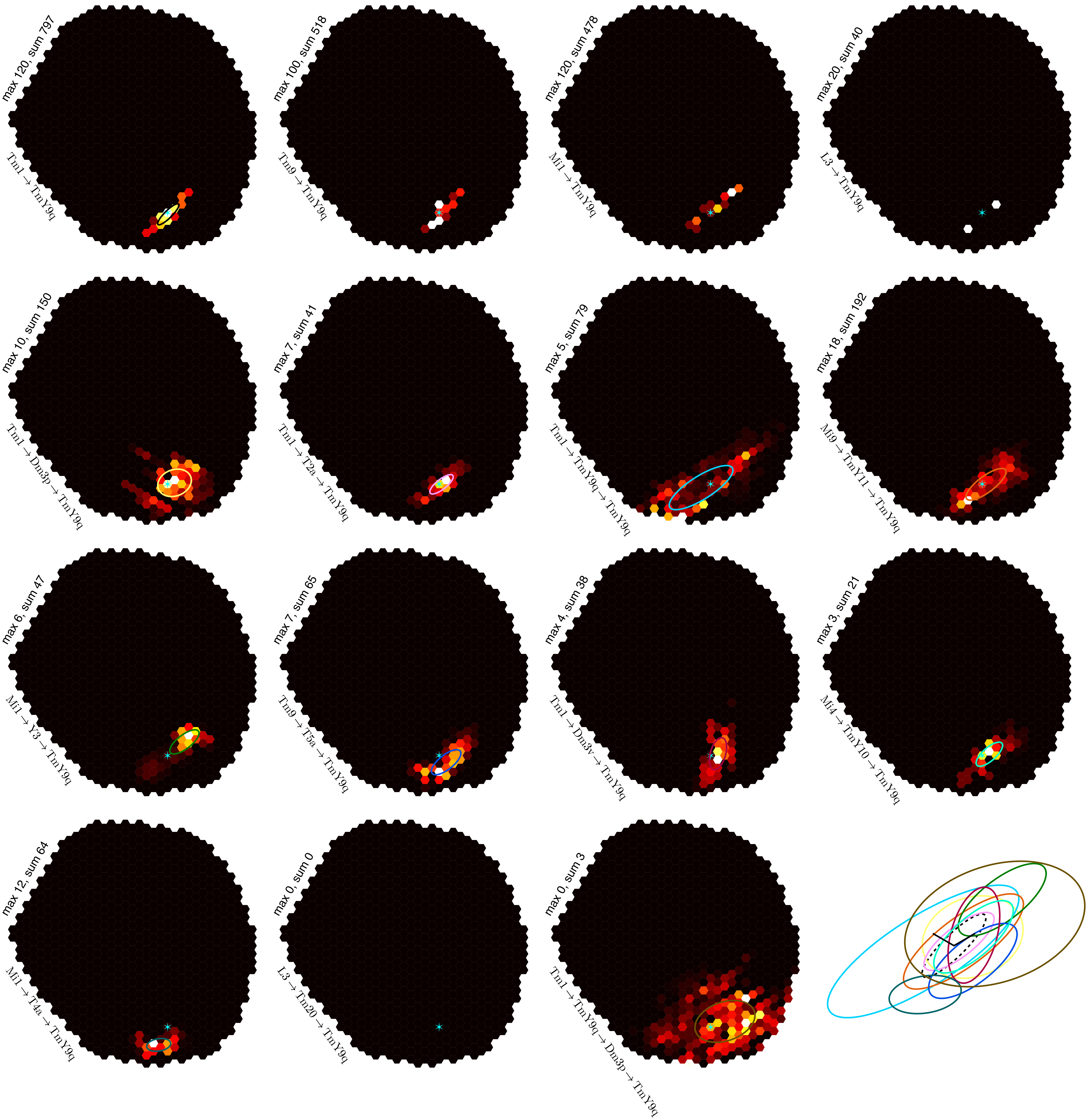
max 120, sum 797
Tm1 → TmY9q
max 100, sum 518
Tm9 → TmY9q
max 120, sum 478
Mi1 → TmY9q
max 20, sum 40
L3 → TmY9q
max 10, sum 150
Tm1 → Dm3p → TmY9q
max 7, sum 41
Tm1 → T2a → TmY9q
max 5, sum 79
Tm1 → TmY9q → TmY9q
max 18, sum 192
Mi9 → TmY11 → TmY9q
max 6, sum 47
Mi1 → Y3 → TmY9q
max 7, sum 65
Tm9 → T5a → TmY9q
max 4, sum 38
Tm1 → Dm3v → TmY9q
max 3, sum 21
Mi4 → TmY10 → TmY9q
max 12, sum 64
Mi1 → T4a → TmY9q
max 0, sum 0
L3 → Tm20 → TmY9q
max 0, sum 3
Tm1 → TmY9q → Dm3p → TmY9q
